# Quality of Life of the Elderly during the COVID-19 Pandemic in Asian Countries: A Cross-Sectional Study across Six Countries

**DOI:** 10.3390/life12030365

**Published:** 2022-03-03

**Authors:** Roy Rillera Marzo, Praval Khanal, Absar Ahmad, Farooq Azam Rathore, Shekhar Chauhan, Akansha Singh, Sunil Shrestha, Ayesha AlRifai, Masoud Lotfizadeh, Delan Ameen Younus, Md. Arif Billah, Farzana Rahman, Yalini Sivaladchanam, Devi Mohan, Tin Tin Su

**Affiliations:** 1 Department of Community Medicine, International Medical School, Management and Science University, Shah Alam 40100, Malaysia; roy.marzo@monash.edu; 2 Department of Community Medicine, Faculty of Medicine, Asia Metropolitan University, Johor Bahru 81750, Malaysia; 3 Global Public Health, Jeffrey Cheah School of Medicine and Health Sciences, Monash University Malaysia, Bandar Sunway 47500, Malaysia; devi.mohan@monash.edu (D.M.); tintin.su@monash.edu (T.T.S.); 4 Department of Health and Ageing, Nepal Health Research and Innovation Foundation, Lalitpur 23513, Nepal; 5 Department of Community Medicine, Manipal Tata Medical College, Manipal Academy of Higher Education, Jamshedpur 831017, India; absar.ahmad@manipal.edu; 6 Department of Rehabilitation Medicine, Armed Forces Institute of Rehabilitation Medicine (AFIRM), Rawalpindi 46000, Pakistan; farooqrathore@amcolians.org; 7 Department of Family and Generations, International Institute for Population Sciences, Mumbai 400001, India; shekhar2486@iips.net; 8 Durham Research Methods Centre, Department of Anthropology, Durham University, Durham DH1 3LE, UK; akansha.singh@durham.ac.uk; 9 School of Pharmacy, Monash University Malaysia, Bandar Sunway 47500, Malaysia; sunil.shrestha@monash.edu; 10 Institute of Community and Public Health, Birzeit University, Birzeit P.O. Box 14, Palestine; a.alrifai1@gmail.com; 11 Department of Community Health, Shahrekord University of Medical Sciences, Shahrekord 88157-13471, Iran; m.lotfizadeh@skums.ac.ir; 12 General Directorate for Scientific Research Center, Salahaddin University-Erbil, Erbil 44001, Iraq; delan.younus@su.edu.krd; 13 Faculty of Business, Economic and Social Development, Universiti Malaysia Terengganu, Kuala Nerus, Kuala Terengganu 21030, Malaysia; p4133@pps.umt.edu.my; 14 Bangladesh National Nutrition Council, Dhaka 1000, Bangladesh; suasha.farzana@gmail.com; 15 Department of Pharmacy, Faculty of Allied Health Sciences, University of Peradeniya, Kandy 20400, Sri Lanka; p45@nmra.gov.lk; 16 South East Asia Community Observatory (SEACO), Jeffrey Cheah School of Medicine and Health Sciences, Monash University Malaysia, Bandar Sunway 47500, Malaysia

**Keywords:** quality of life, older people, COVID-19, mental health, physical health

## Abstract

Background: Few studies have explored the determinants of health-related quality of life (HRQoL) in the elderly during the COVID-19 pandemic. Identifying these factors may help implement appropriate policies to enhance HRQoL in the elderly. Therefore, we aimed to identify the predictors of physical and mental component summary (PCS and MCS) scores of HRQoL in selected six low- and middle-income Asian countries. Methods: We conducted an online survey of older people aged ≥55 years in six countries: Bangladesh, Iran, Iraq, Malaysia, Palestine, and Sri Lanka. The Stark QoL questionnaire was used to measure the PCS and MCS scores. Univariate and multiple variable analyses after adjusting for confounders were performed to identify the possible predictors of PCS and MCS. Results: A total of 1644 older people (69.1 ± 7.8 years, range 55–97 years, Female: 50.9%) responded to the survey. We documented age, country of residence, marital status, number of male children, current employment status, and health insurance, ability to pay household bills, frequency of family members visits and receiving support during COVID-19 pandemic predicted both PCS and MCS. However, gender, residence, and number of female children were associated with PCS only (all *p* < 0.05). Conclusion: Socio-demographic factors such as age, country of residence, marital status, number of male children, current employment status, health insurance, ability to pay household bills, frequency of family members visiting family members, and receiving support during the COVID-19 pandemic affecting both physical and mental quality of life. These results can guide formulating health care planning policies to enhance QoL during COVID-19 and future pandemics in the elderly.

## 1. Introduction

The global population of older people (60+ years) has increased from 382 million in 1980 to 962 million in 2017 and is further expected to rise to 2.1 billion by 2050 [1]. Nearly two-thirds of the world’s older people reside in developing regions. The increase in the elderly population in the lower- and middle-income countries (LMICs) is more rapid than in developed countries [1]. As a result, older people are highly vulnerable to the impact of COVID-19 [2,3,4] and evidence suggests that their quality of Life (QoL) during the pandemic has significantly worsened.

Due to multiple physical, social, and psychological issues, many elderlies are less mobile than young people. The COVID-19 pandemic with resulting lockdowns and instructions for social distancing has restricted nearly everyone to their homes for varying periods. However, the impact is more pronounced on the elderly [5]. This has resulted in a higher risk of various severe, even life-threatening, physical and mental health conditions and a poor quality of life [6,7]. In addition, there are several reports that older people face a challenging situation to manage their mental health [8]. Many of them are struggling with loneliness, social isolation, and prolonged grief as the result of separation from their loved ones [9,10]; resulting in psychological distress [11]; and a poor quality of life [12].

There is increasing evidence that COVID-19 has adversely affected the QoL in different age groups and professions. However, most of these studies have been conducted in the West and developed countries [13,14]. There is a lack of studies on the QoL in the elderly residing in the LMICs in the COVID setting. Further, there is a dearth of studies examining the relative contributions of individual, family, and other relevant factors in the Asian context [15,16], compared to developed countries [17,18]. Identification and understanding of determinants of QoL among the Asian older adults may help policymakers design and implement appropriate policies and programs to maintain the QoL of older Asians during the pandemic.

We carried out an online questionnaire for a survey in six Asian countries to explore the role of age, gender, socioeconomic status, and other family-related predictors on the quality-of-life outcomes.

## 2. Methods

### 2.1. Setting and Design

A cross-sectional survey was conducted among older people aged ≥55 years residing in six LMIC Asian countries (Bangladesh, Iran, Iraq, Malaysia, Palestine, and Sri Lanka). The questionnaire was developed by investigators using Google forms and was distributed using personal contacts and via word of mouth using emails and messenger Apps such as WhatsApp, Telegram, and other social media such as Facebook and Twitter during the first wave of the COVID-19 pandemic.

The first part of the questionnaire was an informed consent form explaining the voluntary participation and confidentiality of the data. Data collection started on 25 March 2020, two weeks after the announcement by the WHO that COVID-19 was a pandemic. The online link was available for two months. The participants were asked to respond only once, and the form settings allowed only one response per user.

### 2.2. Ethical Approval

The study was designed and conducted in accordance with the Helsinki declaration and was approved by the Ethics Committee of Asia Metropolitan University, Malaysia (Ethics Reference Number AMU/MREC/NF/08/032020). All participants completed an informed written consent electronically before completing the survey questionnaire.

### 2.3. Study Questionnaire/Tool

We used the Checklist for Reporting Results of Internet E-Surveys (CHERRIES) guideline to ensure the quality and validity of our online survey (Eysenbach, 2004). We measured QoL using the modified Stark QoL questionnaire. It is a widely used and validated picture-based questionnaire [19], which consists of short questions translated into easy-to-understand sign language and descriptive pictures as response options [20]. It evaluates the perceived QoL using nine mental domain (3 items) and physical domain (6 items). The English version of the instrument was validated in a previous study using the internet [19]. The tool has high reliability for physical and mental components (Cronbach’s alpha 0.93 and 0.63, respectively). The instrument also shows a good construct validity. Scoring, therefore, based on the response options (Table 1), total scores range from 9 to 39. A higher score indicates poor is the quality of life. We used the English version of the Stark QoL, and internal consistency was assessed using Cronbach’s α. The internal reliability of the present study was found to be 0.86. The internal reliability of mental and physical components was 0.57 and 0.87, respectively.

On the first page, three items measuring mental domain are presented. The first item measured mood and consisted of five facial expressions; at one end was a very happy face and a very sad one at another end, with a happy to sad expression in between. Participants were asked to check the one that best applied to them. The second item measured energy and presented two pictures of a person walking; on the left-hand side, the walker was full of energy, and on the right, he seemed to be walking almost as if depressed. The third item measured social contact and displayed three pictures showing a group of five persons each, one depicted as white and four of them grey. The person depicted in white color symbolizes the participant themselves; the grey ones depict a possible peer group. On one end, the person depicted in white person stands in the middle of the group and stands alone on the other end. Together, these three items constituted the mental component. All items were displayed on one page, and participants were asked to choose their answer by making a cross under the picture that best applies to one’s situation.

On the second page, six items that measure physical functioning are presented. The pictures showed activities like carrying a shopping basket, moving a table, tying shoes, etc. Next to each picture, a five-point Likert scale is displayed. The text reads “I can”, and “++” stands for “very well”, “ + ” for “well”, “0” for “fairly”, “-” for “poorly”, and “--” for very poorly. In the analysis, ++ coded as 1, similarly + = 2, 0 = 3, - = 4, -- = 5. Participants were asked to indicate how easily they could perform the activity displayed in each picture. These items constituted the physical component.

### 2.4. Independent Variables

The Physical Component Summary (PCS) and Mental Component Summary (MCS) scores were calculated as described in the methods above. The independent factors considered for the current study were the age of participants, country of residence (Sri Lanka, Bangladesh, Iran, Iraq, Malaysia, and Palestine), gender (male and female), place of residence (refugee camp, rural, urban), marital status (living with spouse and other which includes widow/unmarried/separated), number of male children, number of female children, number of children living with them at present, education level (primary school, high school, Bachelor degree, and Masters/PhD), present employment status (No and Yes), insurance (No and Yes), ability to pay household bills (No and Yes), frequency of family member (s) visiting them (once every week, once every two weeks, once a month, once every two months, or once a year) and getting support during COVID-19 (not at all, very little, rarely, sometimes, and always).

### 2.5. Statistical Analysis

Statistical Package for Social Sciences (SPSS) version 27.0 for windows was used to analyze the data. The continuous variables were expressed as means, standard deviations, minimums, and maximums, while categorical variables were expressed as proportions and frequencies.

Univariate analyses were performed to identify the possible significant factors for the PCS and MCS score. Pearson correlation coefficient was calculated to investigate the correlation for quantitative variables (e.g., age, number of male children, and number of female children). Similarly, an independent sample *t*-test was performed for two groups comparisons, and analysis of variance (ANOVA) was performed for multiple group comparisons to identify the associated variables (the main effect was reported). For the independent *t*-test, effect size was reported in terms of Cohen’s d value, while eta squared was used to report for ANOVA test. Only the significant variables were incorporated into the final linear regression, and the factors associated with physical and mental QoL were identified. A *p*-value of <0.05 was considered statistically significant in all the analyses.

## 3. Results

Out of 1812 responses received, the present study included only 1644 valid questionnaires. Responses from one hundred sixty-eight respondents were excluded due to failing to meet our inclusion criteria (such as participants younger than 55 years) and incomplete data. PCS and MCS scores in our study were 17.9 (5.7) and 7.5 (1.9) (mean (SD)), respectively. The age range of participants was 55–78 years, with a mean age of 69.1 (7.8) years. The frequency distribution of socio-demographic characteristics of participants is shown in Table 2.

Continuous variables are expressed in mean (SD), whereas categorical variables are presented as numbers (percentage). Qualitative and quantitative differences between two groups were analyzed by χ^2^ test for categorical parameters and Student’s *t*-test or ANOVA for continuous parameters

With univariate analyses, there were associations of each of the investigated socio-demographic variables with the PCS and MCS scores (shown in Table 2).

Table 3 depicts the result of linear regression analyses. Upon multiple linear regression considering all the significant variables, the present study identified the age, country of residence, gender, place of residence, marital status, number of male and female children, present employment status, insurance, ability to pay household bills, frequency of family members visiting participants and receiving support during COVID-19 pandemic as the factors of PCS and age, country of residence, marital status, number of male children, present employment status, insurance, ability to pay household bills, frequency of family members visiting participants and receiving support during COVID-19 pandemic as the factors of MCS (all *p* < 0.05).

The positively predicting factors of PCS of quality of life included age (B = 0.233, *p* < 0.001), female gender (B = 0.592, *p* = 0.039), number of male children (B = 0.267, *p* = 0.001) and female children (B = 0.230, *p* = 0.002), single marital status (B = 0.750, *p* = 0.016), receiving support during COVID-19 (for very little support (B = 0.979, *p* = 0.017), and for always support (B = 1.221, *p* = 0.011) in reference to no support at all. There was a negative association of insurance and PCS score (B = −0.904, *p* = 0.010), and Iran compared to Sri Lanka (B = −3.132, *p* < 0.001) with PCS score, frequency of family visiting once in a year compared to once in a week (B = −1.414, *p* = 0.036), enough money to pay house bills (B = −1.518, *p* < 0.001) and residing in refugee camp compared to an urban area (B = −1.323, *p* = 0.043). While in multiple linear regression, employment status and education did not predict the PCS score (all *p* > 0.05). Similarly, positively predicting factors of MCS of quality-of-life included age (B = 0.026, *p* < 0.001), number of male children (B = 0.099, *p* < 0.001), receiving very little support during COVID-19 (B = 0.430, *p* = 0.003) compared to no support at all. There were negative associations of insurance and MCS score (B = −0.645, *p* < 0.001), frequency of family member visiting participants once in a month (B = 0.355, *p* = 0.010) compared to once in a week, present employment status (B = −0.290, *p* = 0.015) and enough money to pay house bills (B = −0.685, *p* < 0.001). Compared to Sri Lanka, Iran (B = −0720, *p* = 0.007), Iraq (B = −0.626, *p* = 0.019), Malaysia (B = −1.212, *p* = 0.003) and Palestine (B = −0.530, *p* = 0.040) were negatively associated with MCS. While in multiple linear regression, gender, number of female children, employment status, marital status, education level, frequency of family member visiting participants, and place of residence did not predict the MCS score (all *p* > 0.05).

## 4. Discussion

The adverse impact of the COVID-19 pandemic has been felt by everyone globally. However, it varies depending upon the country of residence, age, health status, social support, and personal resilience. We noticed a lack of research articles focused on QoL in older adults during and after COVID-19. Most of the published research on the assessment of QoL after COVID-19 focuses on other groups, e.g., survivors of COVID-19 [21], health care professionals [22], and a relatively younger population and female gender [23]. We assessed the QoL in the elderly population in six Low Middle-Income Asian countries using an online survey tool that provides some interesting and unique insights. This study focused on the elderly without COVID-19 addresses a critical research gap in the literature.

The majority of the respondents in the study were from Iran, Iraq, Palestine, and Bangladesh. All of these have predominantly Muslim populations and are considered Eastern societies. The elderly command a special status of respect and authority both in Islamic [24] and Eastern cultures. Despite their age and health issues that accompany aging in these societies, they usually act as heads of the families, enjoy authority, and closely knit with their children. This is reflected in this study where most (87.8%) of the respondents answered that their children lived in the same area. However, the repeated, strict, and prolonged lockdowns in many countries have affected the socialization and frequent contact with relatives and friends. Although we cannot establish a clear causality, these reduced interactions due to the COVID-19 related restrictions might be responsible for more than half of the respondents in the study reporting that they rarely received support or had little to no support at all during the COVID-19. This can adversely affect the mental and psychological health, leading to a reduced QoL.

We need to consider that adverse physical and mental effects of COVID-19 on the QoL have only not been restricted to the elderly. These have been reported in various age groups from different parts of the world, including China, Chile, Vietnam, Turkey, Qatar, Europe, and North America. Shah and colleagues conducted a prospective cross-sectional global online survey using an anonymous online questionnaire [25]. They targeted adults diagnosed with COVID-19 and their family members or partner who could read and understand English. The study respondents reported a major persisting impact on their physical and psychosocial health. The lives of their partners and other family members were also severely affected.

Qi et al. (2020) explored physical activity participation, health-related QoL, and perceived stress among Chinese adults during the COVID-19 pandemic [26]. Although the respondents mean age in their study (31.8) was significantly different from our study (69.1 years), the results were important and comparable to our study population as well [26]. For example, those who were married and had a better education reported significantly lower levels of perceived stress than the single/divorced and those with lower educational status [26]. Similarly, people with lower family incomes had higher levels of perceived stress [26]. This is similar to the results of the current study. They also reported that home confinement led to reduced physical activity and potentially contributed to increased stress and a poor QoL. This is especially important for the old population, who are already less mobile and physically inactive than the younger population.

Engaging in physical activity has many physical and psychological health benefits. There is evidence that physically active people report significantly higher HRQoL and lower levels of perceived stress. In addition, regular exercise has multiple physical and mental health benefits, including the body’s ability to combat infections. Unfortunately, mobility and physical activity levels have slowed during the pandemic lockdown and stay-at-home restrictions. This is particularly alarming for the elderly, most of whom were not active and mobile even before the pandemic for various reasons [26]. In the wake of the pandemic, people confined to their homes spend more time online and on social media, further reducing physical activity levels. Therefore, everyone, particularly the elderly, must be encouraged to engage in regular physical activity and exercises to maintain their physical and mental health. In the meantime, we also identified that social support and frequent family visit has positive impact on QoL among the elderly.

Sepúlveda-Loyola (2020) reviewed the impact of social isolation during the COVID-19 pandemic on the mental and physical health of older people and provided recommendations for patients, caregivers, and health professionals [27]. They recommended physical activity in any form, following a regular sleep–wake cycle, providing mental and psychological health support to the elderly during periods of isolation, cognitive stimulation (either by using apps or stimulating mental exercises, especially in those people with previous cognitive impairments), remaining connected to relatives, reducing exposure to social media, and establishing helplines for the elderly [27]. Physical activity in the elderly during the COVID-19 lockdown in Spain was associated with higher resilience and lower depression. It is essential, that all forms of physical activities and exercises be encouraged for this group of people.

COVID-19 has adversely affected the finances of governments and individuals. While the young ones can support themselves by engaging in different activities, it might be difficult for the elderly to support themselves during this crisis. This is reflected by those employed and having enough money to pay the bills reporting a better QoL in this study.

### Strengths and Limitations

The results of this study should be interpreted in light of several limitations. First, responder bias could be one of the limitations of the study. Data were collected using the snowballing method, which could have affected the heterogeneity of the sample. Therefore, the causality should be made cautiously as reported PCS, and MCS scores may not entirely be related only to the COVID-19 situation. Finally, although the sample was drawn from multiple LMICs it was not uniformly distributed, with Sri Lanka and Malaysia being the least represented.

Furthermore, the data on quality of life, perceived social support, or depression status before the COVID-19 outbreak were not available for comparison. Another limitation is that a large proportion of the sampled population was highly educated, responded to a questionnaire in the English language, and resided in urban areas. This is not truly representative of the general population in these LMIC, which is usually less educated and reside mainly in the rural areas. The strengths of the study include comprehensive data collection from six countries representing a diversity of Asian cultures and societies. The sample size was large enough to draw conclusions and recommendations. In addition, to the best of our knowledge and literature search, this is the first study examining the quality of life in the elderly in a multi-country LMIC setting.

## 5. Conclusions

In this multi-country online survey on the assessment of QoL in the elderly, we found that age, country of residence, marital status, number of male children, current employment status, health insurance, ability to pay household bills, frequency of family members visiting family members, and receiving support during COVID-19 pandemic affecting both physical and mental quality of life. The data were merged from six countries, and the findings observed in the current study suggest that multiple factors need to be considered, and attention should be given to those affecting factors by the younger population, the so-called future elderly, to enhance their QoL in their elderly stage. All of these highlights the role of providing physical, emotional, and psychological support to the elderly who might be having pre-existing physical and mental health issues due to aging. These findings can help formulate health care planning policies to address issues with the QoL during COVID-19 and future pandemics by the policymakers.

## Figures and Tables

**Table 1:** Description of the Outcome variables.

Variable	Description	Constructed Variable
1. Mood	The first item consists of five smileys, at one end is a very happy face, at the other end a very sad one	1 = very good, 2 = Good, 3 = Neutral, 4 = sad, 5 = very sad
2. Energy	The second item presents three pictures of a person walking, on the left-hand side, the walker is full of energy, and on the right, he seems to be walking as if depressed	1 = Full of energy, 2 = Neutral, 3 = No energy
3. Contacts of others	The third item displays three pictures of a group of five persons each, one white and four grey.	1 = Many friends, 2 = limited friend, 3 = no friend
My food during COVID-19 pandemic	My food during COVID-19 pandemic	1 = Eat very well, 2 = eat less, 3 = eat very little
Sweeping up	Sweeping up	1 = Very well, 2 = well, 3 = fairly, 4 = poorly, 5 = very poorly
Jogging	Jogging	1 = Very well, 2 = well, 3 = fairly, 4 = poorly, 5 = very poorly
Moving a table	Moving a table	1 = Very well, 2 = well, 3 = fairly, 4 = poorly, 5 = very poorly
Tying shoes	Tying shoes	1 = Very well, 2 = well, 3 = fairly, 4 = poorly, 5 = very poorly
Lifting a heavy object	Lifting a heavy object	1 = Very well, 2 = well, 3 = fairly, 4 = poorly, 5 = very poorly

**Table 2:** Socio-demographic characteristics and differences in physical and mental component summary scores across subgroups (*n* = 1644).

Variables		PCS			MCS		
	*n* (%)	Mean (SD)	*p*	Effect Size	Mean (SD)	*p*	Effect Size
	Mean (SD)						
Age	Mean age (SD)	69.1 (7.8)	<0.001	r = 0.383	7.50 (1.87)	<0.001	r = 0.152
	1644 (100)						
Country							
Sri Lanka	59 (3.6)	19.08 (5.61)			8.10 (1.41)		
Bangladesh	152 (9.2)	19.63 (5.60)			8.01 (1.71)		
Iran	461 (28.0)	16.08 (5.84)	<0.001	0.046	7.20 (1.88)	<0.001	0.029
Iraq	412 (25.1)	18.79 (5.09)			7.80 (1.80)		
Malaysia	28 (1.70)	17.32 (5.38)			6.86 (1.78)		
Palestine	532 (32.4)	18.27 (5.86)			7.34 (1.95)		
Gender							
Male	837 (50.9)	17.19 (5.74)	<0.001	0.266	7.31 (1.86)	<0.001	0.204
Female	807 (49.1)	18.70 (5.63)			7.69 (1.86)		
Place of residence							
Refugee camp	84 (5.1)	19.17 (4.83)			7.25 (1.46)		
Rural	451 (27.4)	18.40 (5.81)	0.007	0.006	7.65 (1.91)	0.082	0.003
Urban	1109 (67.5)	17.64 (5.74)			7.45 (1.88)		
Marital status							
Living with spouse	1163 (70.7)	17.21 (5.59)	<0.001	0.436	7.37 (1.85)	<0.001	0.23
Widow/Unmarried/Separated	481 (29.3)	19.66 (5.68)			7.80 (1.89)		
Number of female children	-	2.72 (1.87)	<0.001	r = 0.168		0.298	r = 0.026
Number of male children	-	2.74 (1.77)	<0.001	r = 0.163		0.003	r = 0.074
Children living same area							
None	201 (12.2)	17.84 (5.81)	0.681	0.001	7.56 (1.85)	0.002	0.009
One of them	376 (22.9)	18.24 (5.54)			7.74 (1.79)		
Two of them	364 (22.1)	17.76 (5.92)			7.59 (1.86)		
More than two	703 (42.8)	17.87 (5.71)			7.30 (1.91)		
Education							
Primary School	856 (52.1)	18.73 (5.85)			7.61 (1.87)		
High School	371 (22.6)	17.63 (5.29)	<0.001	0.027	7.48 (1.81)	0.012	0.007
Bachelor degree	345 (21.0)	16.72 (5.48)			7.34 (1.87)		
Master/PhD	72 (4.4)	15.67 (5.98)			6.97 (2.05)		
Present Employment							
No	1282 (78.0)	18.50 (5.62)	<0.001	0.466	7.62 (1.85)	<0.001	0.309
Yes	362 (22.0)	15.88 (5.66)			7.05 (1.88)		
Insurance							
No	684 (41.6)	19.28 (5.25)	<0.001	0.412	8.01 (1.74)	<0.001	0.479
Yes	960 (58.4)	16.96 (5.86)			7.13 (1.86)		
Enough money to pay for household bills							
No	552 (33.6)	19.13 (5.64)	<0.001	0.32	7.89 (1.84)	<0.001	0.319
Yes	1092 (66.4)	17.32 (5.68)			7.30(1.86)		
Frequency of family member visit you							
Once every week	616 (37.5)	18.40 (5.64)	0.069	0.005	7.52 (1.83)	<0.001	0.014
Once every two weeks	608 (37.0)	17.76 (5.81)			7.25 (1.87)		
Once a month	237 (14.4)	17.74 (5.70)			7.85 (1.88)		
Once every two months	119 (7.2)	17.30 (5.55)			7.67 (1.95)		
Once a year	64 (3.9)	16.83 (6.09)			7.94 (1.93)		
Received support during the COVID-19 pandemic							
Not at all	274 (16.7)	17.59 (5.87)			7.34 (1.84)		
Very little	330 (20.1)	18.90 (5.13)	<0.001	0.021	7.91 (1.81)	<0.001	0.017
Rarely	501 (30.5)	17.67 (5.41)			7.49 (1.74)		
Sometimes	340 (20.7)	16.84 (5.85)			7.19 (1.94)		
Always	199 (12.1)	19.28 (6.54)			7.56 (2.09)		

**Table 3:** Regression analyses showing factors of PCS and MCS of Quality of Life.

Possible Factors	PCS		MCS	
	B (95% CI)	*p*	B (95% CI)	*p*
Age	0.233 (0.198, 0.268)	<0.001	0.026 (0.014, 0.038)	<0.001
Country				
Sri Lanka	Reference		Reference	
Bangladesh	0.477 (−1.076, 2.030)	0.547	−0.386 (−0.940, 0.168)	0.172
Iran	−3.132 (−4.607, −1.657)	<0.001	−0.720 (−1.246, −0.0194)	0.007
Iraq	−0.777 (−2.239, 0.685)	0.297	−0.626 (−1.147, −0.105)	0.019
Malaysia	−2.124 (−4.386, 0.137)	0.066	−1.212 (−2.019, −0.405)	0.003
Palestine	−0.075 (−1.496, 1.345)	0.917	−0.530 (−1.037, −0.023)	0.04
Gender				
Male	Reference		Reference	
Female	0.592 (0.031, 1.153)	0.039	0.198 (−0.003, 0.398)	0.053
Place of residence				
Urban	Reference		Reference	
Refugee camp	−1.324 (−2.609, −0.039)	0.043	−0.602 (−1.060, −0.144)	0.01
Rural	−0.273 (−0.868, 0.322)	0.369	−0.005 (−0.217, −0.207)	0.964
Marital status			Reference	
Living with spouse	Reference			
Widow/Unmarried/Separated	0.750 (0.139, 1.362)	0.016	0.071 (−0.148, 0.289)	0.526
Number of female children	0.230 (0.086, 0.374)	0.002	0.018 (−0.033, 0.069)	0.496
Children living same area				
None	Reference		Reference	
One of them	−0.078 (−0.960, 0.803)	0.862	0.130 (−0.184, 0.444)	0.417
Two of them	0.006 (−0.901, 0.913)	0.989	0.155 (−0.168, 0.479)	0.347
More than two	−0.545 (−1.441, 0.352)	0.233	−0.159 (−0.479, 0.160)	0.328
Education				
Primary School	Reference		Reference	
High School	−0.090 (−0.761, 0.580)	0.792	0.096 (−0.143, 0.335)	0.432
Bachelor degree	−0.021 (−0.771, 0.730)	0.957	0.259 (−0.009, 0.527)	0.058
Master/PhD	−0.142 (−1.479, 1.196)	0.836	0.024 (−0.453, 0.501)	0.921
Present Employment				
No	Reference		Reference	
Yes	−1.279 (−1.932, −0.626)	<0.001	−0.290 (−0.523, −0.057)	0.015
Insurance				
No	Reference		Reference	
Yes	−0.904 (−1.587, −0.221)	0.01	−0.645 (−0.889, −0.402)	<0.001
Enough money to pay for household bills				
No	Reference		Reference	
Yes	−1.518 (−2.084, −0.952)	<0.001	−0.685 (−0.887, −0.483)	<0.001
Frequency of family member visit you				
Once every week	Reference			
Once every two weeks	−0.381 (−0.0949, 0.187)	0.188	−0.155 (−0.357, 0.048)	0.134
Once a month	−0.331 (−1.090, 0.427)	0.392	0.355(0.085, 0.626)	0.01
Once every two months	−0.077 (−1.071, 0.916)	0.879	0.241 (−0.113, 0.595)	0.182
Once a year	−1.414 (−2.732, −0.096)	0.036	0.362 (−0.108, 0.833)	0.131
Received support during COVID-19 Not at all	Reference		Reference	
Very little	0.979 (0.176, 1.783)	0.017	0.430 (0.143, 0.716)	0.003
Rarely	0.067 (−0.696, 0.831)	0.863	0.132 (−0.141, 0.404)	0.343
Sometimes	−0.259 (−1.076, 0.557)	0.533	−0.062 (−0.353, 0.229)	0.676
Always	1.221 (0.276, 2.165)	0.011	0.259 (−0.078, 0.596)	0.132

## Data Availability

The data used in the present study are available from reasonable request from corresponding author.

## References

[1] United Nations, Department of Economic and Social Affairs, & Population Division (2017). World Population Ageing: 2017 Highlights.

[2] Banerjee D. (2020). Age and Ageism in COVID-19: Elderly Mental Health-Care Vulnerabilities and Needs. Asian J. Psychiatry.

[3] Calderón-Larrañaga A., Dekhtyar S., Vetrano D.L., Bellander T., Fratiglioni L. (2020). COVID-19: Risk Accumulation among Biologically and Socially Vulnerable Older Populations. Ageing Res. Rev..

[4] Wyper G.M., Assunção R., Cuschieri S., Devleesschauwer B., Fletcher E., Haagsma J.A., Hilderink H.B., Idavain J., Lesnik T., Von der Lippe E. (2020). Population Vulnerability to COVID-19 in Europe: A Burden of Disease Analysis. Arch. Public Health.

[5] Lloyd-Sherlock P., Ebrahim S., Geffen L., McKee M. (2020). Bearing the Brunt of COVID-19: Older People in Low and Middle Income Countries. BMJ.

[6] Groarke J.M., Berry E., Graham-Wisener L., McKenna-Plumley P.E., McGlinchey E., Armour C. (2020). Loneliness in the UK during the COVID-19 Pandemic: Cross-Sectional Results from the COVID-19 Psychological Wellbeing Study. PLoS ONE.

[7] Talarska D., Tobis S., Kotkowiak M., Strugała M., Stanisławska J., Wieczorowska-Tobis K. (2018). Determinants of Quality of Life and the Need for Support for the Elderly with Good Physical and Mental Functioning. Med. Sci. Monit. Int. Med. J. Exp. Clin. Res..

[8] Girdhar R., Srivastava V., Sethi S. (2020). Managing Mental Health Issues among Elderly during COVID-19 Pandemic. J. Geriatr. Care Res..

[9] Goveas J.S., Shear M.K. (2020). Grief and the COVID-19 Pandemic in Older Adults. Am. J. Geriatr. Psychiatry.

[10] Grossman E.S., Hoffman Y.S., Palgi Y., Shrira A. (2021). COVID-19 Related Loneliness and Sleep Problems in Older Adults: Worries and Resilience as Potential Moderators. Personal. Individ. Differ..

[11] Wang Y., Fu P., Li J., Jing Z., Wang Q., Zhao D., Zhou C. (2021). Changes in Psychological Distress before and during the COVID-19 Pandemic among Older Adults: The Contribution of Frailty Transitions and Multimorbidity. Age Ageing.

[12] Levkovich I., Shinan-Altman S., Essar Schvartz N., Alperin M. (2021). Depression and Health-Related Quality of Life among Elderly Patients during the COVID-19 Pandemic in Israel: A Cross-Sectional Study. J. Prim. Care Community Health.

[13] Cleaton N., Raizada S., Barkham N., Venkatachalam S., Sheeran T., Adizie T., Sapkota H., Singh B., Bateman J. (2020). COVID-19 Prevalence and the Impact on Quality of Life from Stringent Social Distancing in a Single Large UK Rheumatology Centre. Ann. Rheum. Dis..

[14] Ferreira L.N., Pereira L.N., da Fé Brás M., Ilchuk K. (2021). Quality of Life under the COVID-19 Quarantine. Qual. Life Res..

[15] Van Nguyen T., Van Nguyen H., Nguyen T.D., Van Nguyen T., Nguyen T.T. (2017). Difference in Quality of Life and Associated Factors among the Elderly in Rural Vietnam. J. Prev. Med. Hyg..

[16] Woo J., Chan R., Leung J., Wong M. (2010). Relative Contributions of Geographic, Socioeconomic, and Lifestyle Factors to Quality of Life, Frailty, and Mortality in Elderly. PLoS ONE.

[17] Marengoni A., Angleman S., Melis R., Mangialasche F., Karp A., Garmen A., Meinow B., Fratiglioni L. (2011). Aging with Multimorbidity: A Systematic Review of the Literature. Ageing Res. Rev..

[18] Schorr A.V., Khalaila R. (2018). Aging in Place and Quality of Life among the Elderly in Europe: A Moderated Mediation Model. Arch. Gerontol. Geriatr..

[19] Hardt J. (2015). A New Questionnaire for Measuring Quality of Life-the Stark QoL. Health Qual. Life Outcomes.

[20] Fellinger J., Dall M., Gerich J., Fellinger M., Schossleitner K., Barbaresi W.J., Holzinger D. (2021). Is It Feasible to Assess Self-Reported Quality of Life in Individuals Who Are Deaf and Have Intellectual Disabilities?. Soc. Psychiatry Psychiatr. Epidemiol..

[21] Dorri M., Bazargany M.H.M., Khodaparast Z., Bahrami S., Alan M.S., Rahimi F., Kamipoor Z., Niksima M.M., Dehghan H., Rastad H. (2021). Psychological Problems and Reduced Health-Related Quality of Life in the COVID-19 Survivors. J. Affect. Disord. Rep..

[22] Suryavanshi N., Kadam A., Dhumal G., Nimkar S., Mave V., Gupta A., Cox S.R., Gupte N. (2020). Mental Health and Quality of Life among Healthcare Professionals during the COVID-19 Pandemic in India. Brain Behav..

[23] Schiavi M.C., Spina V., Zullo M.A., Colagiovanni V., Luffarelli P., Rago R., Palazzetti P. (2020). Love in the Time of COVID-19: Sexual Function and Quality of Life Analysis during the Social Distancing Measures in a Group of Italian Reproductive-Age Women. J. Sex. Med..

[24] BBC (2021). What Does Islam Say about Family Life?-Family Life-GCSE Religious Studies Revision. https://www.bbc.co.uk/bitesize/guides/zk9whyc/revision/3.

[25] Shah R., Ali F.M., Nixon S.J., Ingram J.R., Salek S.M., Finlay A.Y. (2021). Measuring the Impact of COVID-19 on the Quality of Life of the Survivors, Partners and Family Members: A Cross-Sectional International Online Survey. BMJ Open.

[26] Qi M., Li P., Moyle W., Weeks B., Jones C. (2020). Physical Activity, Health-Related Quality of Life, and Stress among the Chinese Adult Population during the COVID-19 Pandemic. Int. J. Environ. Res. Public Health.

[27] Sepúlveda-Loyola W., Rodríguez-Sánchez I., Pérez-Rodríguez P., Ganz F., Torralba R., Oliveira D.V., Rodríguez-Mañas L. (2020). Impact of Social Isolation due to COVID-19 on Health in Older People: Mental and Physical Effects and Recommendations. J. Nutr. Health Aging.

